# The principles of tomorrow's university

**DOI:** 10.12688/f1000research.17425.1

**Published:** 2018-12-11

**Authors:** Daniel S. Katz, Gabrielle Allen, Lorena A. Barba, Devin R. Berg, Holly Bik, Carl Boettiger, Christine L. Borgman, C. Titus Brown, Stuart Buck, Randy Burd, Anita de Waard, Martin Paul Eve, Brian E. Granger, Josh Greenberg, Adina Howe, Bill Howe, May Khanna, Timothy L. Killeen, Matthew Mayernik, Erin McKiernan, Chris Mentzel, Nirav Merchant, Kyle E. Niemeyer, Laura Noren, Sarah M. Nusser, Daniel A. Reed, Edward Seidel, MacKenzie Smith, Jeffrey R. Spies, Matt Turk, John D. Van Horn, Jay Walsh

**Affiliations:** 1 Department of Computer Science, University of Illinois at Urbana-Champaign, Urbana, IL, USA; 2 Department of Electrical and Computer Engineering, University of Illinois at Urbana-Champaign, Urbana, IL, USA; 3 National Center for Supercomputing Applications, University of Illinois at Urbana-Champaign, Urbana, IL, USA; 4 School of Information Sciences, University of Illinois at Urbana-Champaign, Urbana, IL, USA; 5 Department of Astronomy, University of Illinois at Urbana-Champaign, Urbana, IL, USA; 6 College of Education, University of Illinois at Urbana-Champaign, Urbana, IL, USA; 7 George Washington University, Washington, DC, USA; 8 Engineering and Technology Department, University of Wisconsin, Stout, Menomonie, WI, USA; 9 Department of Nematology, University of California-Riverside, Riverside, CA, USA; 10 Department of Environmental Science, Policy and Management, University of California, Berkeley, Berkeley, CA, USA; 11 University of California, Los Angeles, Los Angeles, CA, USA; 12 School of Veterinary Medicine, University of California, Davis, Davis, CA, USA; 13 Laura and John Arnold Foundation, Houston, TX, USA; 14 Long Island University, Brookville, NY, USA; 15 Elsevier Research Products, Jericho, VT, USA; 16 Birkbeck University of London, London, UK; 17 California Polytechnic State University, San Luis Obispo, CA, USA; 18 Alfred P. Sloan Foundation, New York, NY, USA; 19 Iowa State University, Ames, IA, USA; 20 University of Washington, Seattle, Seattle, WA, USA; 21 Department of Pharmacology, University of Arizona, Tucson, AZ, USA; 22 Center for Innovation in Brain Science, University of Arizona, Tucson, AZ, USA; 23 University of Illinois, Urbana, IL, USA; 24 National Center for Atmospheric Research, Boulder, CO, USA; 25 Departamento de Física, Universidad Nacional Autónoma de México, Mexico City, Mexico; 26 Gordon and Betty Moore Foundation, Palo Alto, CA, USA; 27 UA Data Science Institute, University of Arizona, Tucson, AZ, USA; 28 School of Mechanical, Industrial, and Manufacturing Engineering, Oregon State University, Corvallis, OR, USA; 29 Obsidian Security, Newport Beach, CA, USA; 30 University of Utah, Salt Lake City, UT, USA; 31 University of California, Davis, Davis, CA, USA; 32 221B, Columbus, OH, USA; 33 USC Mark and Mary Stevens Neuroimaging and Informatics Institute, Keck School of Medicine, University of Southern California, Los Angeles, CA, USA; 34 Northwestern University, Evanston, IL, USA

**Keywords:** open science, open scholarship, academia, reproducibility, data science, credit, education

## Abstract

In the 21st Century, research is increasingly data- and computation-driven. Researchers, funders, and the larger community today emphasize the traits of openness and reproducibility. In March 2017, 13 mostly early-career research leaders who are building their careers around these traits came together with ten university leaders (presidents, vice presidents, and vice provosts), representatives from four funding agencies, and eleven organizers and other stakeholders in an NIH- and NSF-funded one-day, invitation-only workshop titled "Imagining Tomorrow's University." Workshop attendees were charged with launching a new dialog around open research – the current status, opportunities for advancement, and challenges that limit sharing.

The workshop examined how the internet-enabled research world has changed, and how universities need to change to adapt commensurately, aiming to understand how universities can and should make themselves competitive and attract the best students, staff, and faculty in this new world. During the workshop, the participants re-imagined scholarship, education, and institutions for an open, networked era, to uncover new opportunities for universities to create value and serve society. They expressed the results of these deliberations as a set of 22 principles of tomorrow's university across six areas: credit and attribution, communities, outreach and engagement, education, preservation and reproducibility, and technologies.

Activities that follow on from workshop results take one of three forms. First, since the workshop, a number of workshop authors have further developed and published their white papers to make their reflections and recommendations more concrete. These authors are also conducting efforts to implement these ideas, and to make changes in the university system.  Second, we plan to organise a follow-up workshop that focuses on how these principles could be implemented. Third, we believe that the outcomes of this workshop support and are connected with recent theoretical work on the position and future of open knowledge institutions.

## Summary

In the 21st Century, research is increasingly data- and computation-driven. Researchers, funders, and the larger community today emphasize the traits of openness and reproducibility. In March 2017, 13 mostly early-career research leaders who are building their careers around these traits came together with ten university leaders (presidents, vice presidents, and vice provosts), representatives from four funding agencies, and eleven organizers and other stakeholders in an NIH- and NSF-funded one-day, invitation-only workshop titled “Imagining Tomorrow’s University.” Workshop attendees were charged with launching a new dialog around open research – the current status, opportunities for advancement, and challenges that limit sharing.

The workshop examined how the internet-enabled research world has changed, and how universities need to change to adapt commensurately, aiming to understand how universities can and should make themselves competitive and attract the best students, staff, and faculty in this new world. During the workshop, the participants reimagined scholarship, education, and institutions for an open, networked era, to uncover new opportunities for universities to create value and serve society. They expressed the results of these deliberations as a set of 22 principles of tomorrow's university across six areas: Credit and Attribution (A), Open Scholarship Communities (C), Outreach and Engagement (O), Education (E), Preservation and Reproducibility (P), and Technologies (T):

**Credit and Attribution (A)**

A1 Stakeholders (funders, universities, and researchers) should incentivize credit and attribution for a diverse range of research products and activities, such as research software, dissemination, infrastructure, data products and repositories.
A2 Research should be assessed on its own merits, not based on its appearance in exclusive publication venues. This could be through the use of article-level metrics or narrativized impact measures. Journal impact factors should not be used to evaluate researchers.
A3 Institutions that comparatively measure attention scores should evaluate attention measures with care, since such altmetrics may not correlate with measures of quality.
A4 Institutions should provide appropriate career paths for staff working on open research and maintain the institutional infrastructure required for open research, including recognizing and valuing new, emergent forms of digital outcomes, such as software and data creation, curation and preservation, that are crucial to open research endeavors. These pathways may require rethinking existing classifications and assessments of tenure-track, non-tenure-track, and staff categories of university participants, and funder support for personnel in these categories.

**Open Scholarship Communities (C)**

C1 Scholarly communities are both the target and the product of open scholarship and open research. Therefore, the fostering of communities is a key driver for open research.
C2 These communities can take many forms. They may, for example, coalesce around tools, practices, shared interests, shared data or software, or shared hashtags. They can be short- or long-lived, explicitly funded, or emerge organically from shared interests among the participants. All of these variations are valuable components in the open research social ecosystem, and they must be supported as such by multiple resources, including travel funds, virtual networks, compensations for networking events, etc.
C3 Community activities often serve to evaluate, encourage, and improve the use of tools, software, platforms, and data that are critical to open scholarship. All stakeholders must take steps to encourage these communities to develop, such as supporting common standards (and rewarding those who work on them), funding projects that form a “connective tissue” between different communities. They should also actively encourage sharing practices for tools, and people across communities.

**Outreach and Engagement with the Public (O)**

O1 Outreach and engagement, for public access to and understanding of research outcomes, depend on the audience and may encompass products, processes, and dissemination.
O2 Universities and researchers share a mutual interest in interactions with diverse audiences.
O3 Institutions should value, recognize, and support researchers who participate in outreach and engagement activities, crediting them as components of "service" to the institution.
O4 Access to researchers (scholars) builds trust in the products and processes of research (scholarship).

**Education (E)**

E1 Every student should be guided to understand and learn how to access and use data and software to be well prepared for diverse, modern career paths.
E2 Through open research training, universities should play an active role in increasing research by enabling evidence-based decisions, accelerating discovery, and extending impact to broader communities.
E3 Universities should encourage their faculty to engage in open educational practices, including creating and assigning open educational resources, and reflecting open culture in their courses.

**Preservation and Reproducibility (P)**

P1 The scholarly publication and communication ecosystem should support open and reproducible research, and enable credit for these efforts. Universities should encourage these initiatives by creating incentives (e.g., promotion and tenure categories, service recognition) for such activities.
P2 Research funders should support open and reproducible research by making reproducibility part of their merit review criteria. They should also create new scholarly communication venues or support open scholarship efforts, and encourage, require, and reward reproducible research efforts.
P3 Incentives that promote the public sharing and distribution of scholarly knowledge for open/transparent/reproducible research practices must be put in place. Publishers must require, when appropriate, submissions that provide open and reproducible workflows, and embed this requirement in their own workflows.
P4 Universities should recognize the activities of faculty to educate and train researchers on open and reproducible research skills. Global and national bodies (e.g., National Academies) should promote this recognition across universities.

**Technologies (T)**

T1 Open source technologies, tools and platforms provide intrinsic value to researchers and educators and are an effective way of accelerating open scholarship. Academic institutions should favor and encourage open source solutions as much as possible.
T2 A diverse and interoperable set of tools for open research should be known, shared, and clearly documented.
T3 Institutions should provide and support foundational open scholarship infrastructure (technological and human) for all members of campus.
T4 Institutions should recognize the contributions made by all members of campus to open scholarship infrastructure.

Activities that follow on from workshop results take one of three forms. First, since the workshop, a number of workshop authors have further developed and published their white papers to make their reflections and recommendations more concrete. These authors are also conducting efforts to implement these ideas, and to make changes in the university system. Second, we plan to organise a follow-up workshop that focuses on how these principles could be implemented. Third, we believe that the outcomes of this workshop support and are connected with recent theoretical work on the position and future of open knowledge institutions.

## 1 Introduction

The culture of today’s research universities is built from elements of past universities, including the medieval roles and responsibilities of faculty, the 18–19th century structure of departments, and the mid-20th century research funding model. While for most of history education was reserved for elites, educational access in the United States has been repeatedly expanded, beginning with creation of state-funded universities in the 18th century, and the later creation of land-grant institutions in the 19th century. In the 20th century, this democratization of education was accelerated by the post-World War II GI Bill. Today, higher education is seen as necessary for the majority of young adults to succeed in the growing knowledge economy.

Academic research is also changing. Research practices have been evolving rapidly, based largely on advances in computing capability and capacity. Examples include the use of computational and data science as new research methods, and concomitant changes in research culture, such as vastly increased sharing via the Internet, and collaboration via open source software, open science, and more generally, open scholarship. Simply put, research and scholarship are increasingly data- and computation-driven. At the same time, researchers, funders, and the larger community increasingly emphasize the traits of openness and reproducibility. While the idea of the openness has long been a part of research institutions, new technologies amplify the expectations around openness because the general distribution of information, and the creation of virtual/distributed collaborations (e.g. open source communities), are much easier technically via the internet.

In March 2017, in response to this changing environment, 13 early-career research leaders who are building their careers around these traits came together with 10 university leaders (presidents, vice presidents, and vice provosts), representatives of four funding agencies, and 11 organizers and other stakeholders in an NIH- and NSF-funded one-day, invitation-only workshop called “Imagining Tomorrow’s University.” Workshop attendees were charged with launching a new dialog around open research: current status, opportunities for advancement, and challenges limiting sharing.

The workshop addressed the changing nature of research and the associated shifts in university needs. Some guiding questions at the workshop included:

- How should the university change?
- How should it adapt its structure, mission, infrastructure, education, and recruitment plans?
- Do we need new educational programs?
- Do we need new disciplines or new departments?
- How can universities recognize the value in new types of research products such as software and data?
- Does research staffing need to change?
- Do research data engineers or research software engineers have a place in modern scholarship?
- What are different measures of success for faculty active in open science/open research?

These issues can be summarized as seeking to understand how universities can and should maximize their competitiveness in attracting the best students, staff, and faculty, and better serve the public. In the workshop, the participants tried to reimagine scholarship, education, and institutions for an open, networked era, to discover new opportunities for universities to create value and serve society, expressed as a set of principles.

The workshop included 37 participants and two facilitators (list of attendees can be found in the Appendix). The participants worked in six areas:

- credit and attribution,
- communities,
- education,
- outreach and engagement,
- preservation and reproducibility, and
- technologies,

each of which came to consensus on a set of principles. In addition, the participants discussed three cross-cutting topics: libraries, career paths, and campus IT organizations & data repositories.

## 2 Workshop inputs

In total, 12 of the 14 invited university researchers submitted white papers as their inputs to the workshop, as summarized in Section 2.1. In addition, 12 invited university leaders completed a brief survey, with results summarized in Section 2.2. (One university researcher who submitted a white paper and two university leaders who completed the survey did not attend the workshop due to conflicts that arose at the last minute.)

### 2.1 White papers

Berg ^1^ presents personal reflections on the role that open research can play in defining the purpose and activities of the university. He includes specific recommendations on how the public university can recommit and push the boundaries of its role as the creator and promoter of public knowledge, serving a vital role to the continued economic, social, and technological development of society. The recommendations are: requiring that research products be made openly available, supported via institutional repositories and copying policies; converting technology commercialization offices into research impact offices; empowering and funding university libraries to support open knowledge dissemination; and infusing open knowledge dissemination and best practices into education. Berg also includes some thoughts on how this applies specifically to the field of engineering and how a culture of openness and sharing within the engineering community can help drive societal development.

Bik ^2^ defines open science as “any type of scientific research effort that is freely available and publicly accessible.” This includes “both traditional research products (peer-reviewed publications, underlying datasets) as well as non-traditional initiatives and products (blog posts, slide decks, course syllabi and materials, scientific software, as well as analysis scripts and code).” She says that making science open is important because research is mostly taxpayer-funded; open science is more accountable and more reproducible, more democratic and more accessible, and helps to build a scientist’s reputation. Open science also impacts society by making scientists and their work more visible and more accessible to the public and to policymakers, and more visible to each other. She also discusses some challenges: how open scientists can find each other at a university; that open science has a cost in terms of increased work and reporting systems that don’t support it; merit and promotion policies that don’t recognize or reward open science; and a lack of guidelines and support from the university.

Boettiger ^3^ defines open science as “just science without the barriers created by other incentives.” He says open science has four main pillars: open access (including concerns about paywalls and licenses), open data, open code, and open context, all of which he supports in his own research practices via preprints, data publishing, software publishing and containers, and an open lab notebook. He also teaches about open science, and makes his teaching materials open. He believes that there are social challenges in changing the underlying incentive structure for research, technical challenges in developing solutions that help researchers realize the benefits from open science practices rather than just the costs, and educational challenges that if met could develop the next generation of researchers who implement the needed social and technical changes.

Brown ^4^ thinks Twitter and blogging are integral to the pursuit of open science, and he blogged answers to the workshop questions, including defining open science both as “the philosophical perspective that sharing is good and that barriers to sharing should be lowered as much as possible” and as the practice of lowering the barriers. He says that open science should drive science forward faster, increase its societal impact, open opportunities for serendipity, and “aid with reproducibility and replication, decrease the effects of economic inequality in the sciences by liberating ideas from subscription paywalls, and providing reusable materials for teaching and training.” He believes that “while most scientists are supportive of open science in theory, in fact most scientists are leery of actually sharing things widely before publication,” because of the incentive systems in place. He says that prominent senior researchers need to “visibly and loudly abandon the broken ‘journal prestige’ system, forcefully push back against university administration on matters of research evaluation and tenure, and be a loud presence on grant panels and editorial boards.”

Eve ^5^ says that while for published research work to be “open,” it must be free to read online and free to re-use, open research also concerns the practices of academia opening itself to inspection and collaboration in new ways. Without research being open, research institutions such as universities are not “woven into the tapestry of modern citizenship,” researchers cannot fully pursue new knowledge, and replication and rigor are problematic. Eve is the youngest full professor of English Literature in the UK, and he believes this is due in part to his service and charitable activities, such as being a founder and co-CEO of the Open Library of Humanities, a charitable/not-for-profit open access publishing company that funds or publishes 27 fully open access, zero-fee-for-authors journals. It is supported on an ongoing basis by an international consortium of over 220 (and growing) academic libraries. He describes a range of social, technical, and economic challenges in the implementation of open research, and suggests a set of university changes that could address them, including: not using journal-level or press-level metrics, policies that promote open research practice, strong local green open access policies, and university presses moving to open access as dissemination vehicles.

Howe *et al*. ^6^ believe that “increased transparency in the scientific process can broaden and deepen scientific inquiry, understanding, and impact,” but that this is not quick, effortless, or easy. They propose “that open science can most effectively enable this evolution when it is conceptualized as a multifaceted pathway that includes: the provision of accessible and well-described data, along with information about its context, the methodology and mechanisms necessary to reproduce data analyses, and training products that provide transparent understanding of how the data can be applied to answer questions.” They suggest that doing this often requires investments by researchers, and that changes should be carefully planned across the entire university to avoid unintended consequences.

Howe and Grechkin ^7^ consider open science to be, “a movement to bring the incentives that drive science back in line with the stated values of science,” practices, norms, and tools that reward the sharing of knowledge in addition to the creation of knowledge. They believe that this movement is progressing, but also suggest considering an alternative, “a more transformative vision for wide open science.” Wide open science means 1) wide open experiments, where each experiment consists of a pre-registered hypothesis, a visualization of the result, enough text to interpret and understand it, and the code, data, and environment needed to recreate it; 2) wide open data, supported by tools that automatically curate, integrate, clean, and standardize available data for reuse and reproducibility; and 3) wide open publishing, where overlay journals superimpose a journal-like structure on open access materials to “reduce publication friction while enabling community-driven peer review and curation.”

Khanna ^8^ defines open science as “the dissemination of research in any open forum, publicly available for all to access.” She believes that this will improve research and learning, ensure transparency, and foster more collaborative research. She suggests that, “it is our duty and moral obligation to inform the public about the work being done in our research laboratories,” in part to generate interest and maintain funding. However, to do this, for example, to publish data in an open forum, takes time away from traditional research, teaching, and service. Tenure evaluation often relies mainly on publications and funding, and open science may not be seen as a contribution. If openness were rewarded within the tenure process, perhaps as an expected part of research, teaching, and service, it would increase.

Mayernik ^9^ suggests that “the movements by national governments, funding agencies, universities, and research communities toward ‘open data’ face many difficult challenges.” He believes that this is because, “researchers” data and metadata practices are expected to be robust and structured,” but that they are not. This is in part because researchers are expected to be good at research, not good at depositing data or creating metadata, and because, “making data open in a transparent way can involve a significant investment of time and resources with no obvious benefits [to the researcher].” He relates the concepts of accountability and transparency with researcher actions, and suggests that achieving them is an ongoing process, not the results of one-time acts.

McKiernan ^10^ discusses open scholarship, such as the sharing of articles, code, data, and educational resources, as having the potential to improve or even transform university research and education, and to increase the external impact of universities. She presents numerous case studies and her own personal experiences as a practicing open scholar. Tension is created by incompatibilities between institutional policies and personal practice in many forms of academic evaluation. She proposes actions universities could take to support open scholarship, and explains their benefits. She says, “I do not think most of these actions would require new funding, but rather a redistribution of existing funds and a rewriting of internal policies to better align with university missions of knowledge dissemination and societal impact.”

Niemeyer ^11^ defines open research as “the activity of performing scientific research in a manner that makes products and findings accessible to anyone. This includes sharing data openly (open data), publicly releasing the source code for research software (open source software), and making the written products of research openly accessible (open access).” He believes it is important because of six benefits it supports: accessibility, reproducibility, impact, establishing priority, encouraging trust, and being nice. He has created an open policy for his group’s research. Niemeyer says, “the challenges impeding greater adoption of open science practices are mainly institutional and cultural, rather than technical,” and he makes four recommendations for universities to overcome the challenges: 1) Tenure and promotion should consider the accessibility/openness of research products along with their quantity and “quality”; 2) recognize research products such as software and data as equal to traditional publications in scholarly impact; 3) recognize that publishing in traditional venues may hinder openness, so reduce their importance for promotion and tenure; and 4) support efforts to teach undergraduate and graduate students open science skills, and those necessary to work with software and data, with the same enthusiasm that traditional lab courses receive.

Sengupta and Shanahan ^12^ talk about opening the practice and ongoing work of science, rather than its products. They suggest that we engage “the public in the dynamic, conceptual and representational work involved in creating scientific knowledge.” They propose “public computing spaces, a genre of open-ended, public learning environment where visitors interact with open source computing platforms to directly access, modify and create complex and authentic scientific work” as a possible model of open science in the university.

Some common themes of all white papers are:

- Open scholarship is perhaps the most broad term we can use; it includes open science, open humanities, and open research, and can be defined as opening products such as articles, data, software, educational resources, or more broadly, opening the process of scholarship.
- Costs and benefits for scholars:
  – Researchers respond to how they are evaluated, which today mostly does not reward open scholarship.
  – Sharing can lead to increased progress and knowledge, but can have a cost when it is not rewarded.
- Benefit to society:
  – Openness can reduce the negative image of the university as an ivory tower.
  – Most research funding comes from the public, and they should be given access to the research outputs that they have supported.
  – If we can involve the public in the whole process and not just the outputs, they may have more appreciation of scholarship and the scientific process.
- While parts of the university have the ostensible goal of disseminating research (e.g., university press, technology transfer office), they are often siloed into centers that are measured on financial return.

### 2.2 Leader survey

The results of the survey given in advance of the workshop to the university leaders in attendance are presented next.

1. To what extent is research at your university becoming substantially more data- and computation-driven?

**Figure 1.**
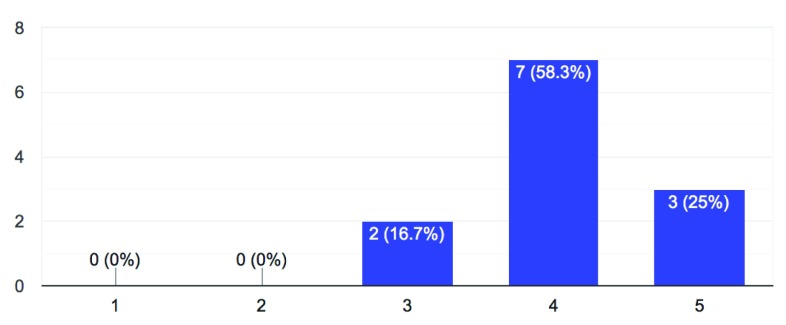
Increase in data- and computation-driven research (1 = minimal change – 5 = substantial change).

2. How important are the open science themes of sharing and reproducibility to your university’s researchers?

**Figure 2.**
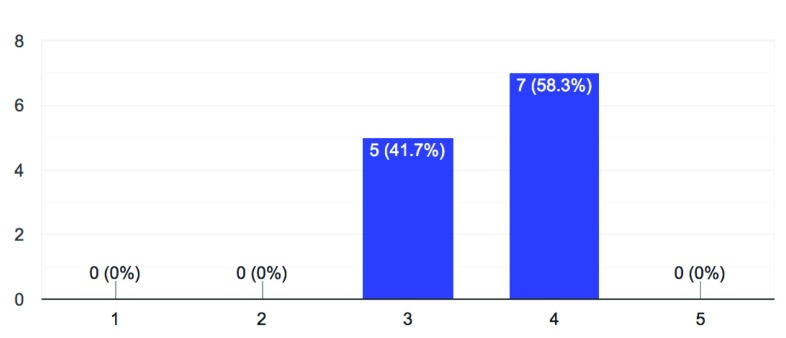
Importance of sharing and reproducibility (1 = not important – 5 = very important).

3. How much investment is your university making to integrate open science into the research environment and curriculum?

**Figure 3.**
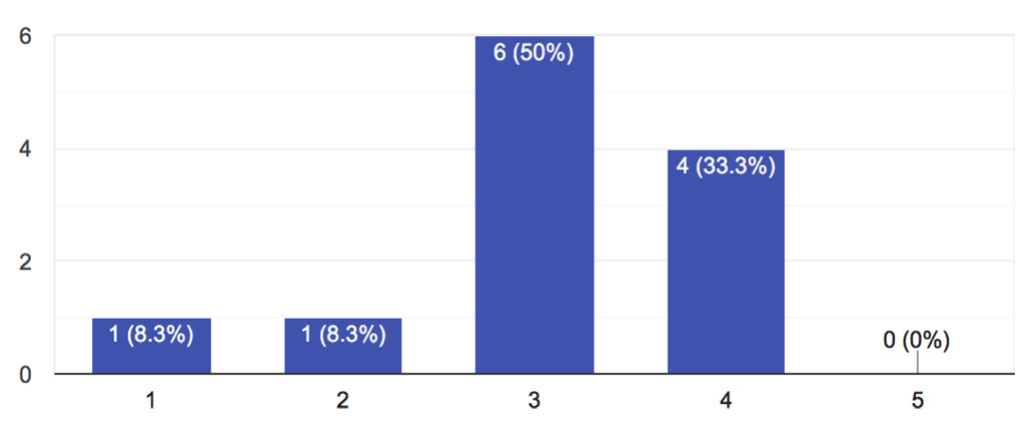
Investment in integrating open science (1 = minimal – 5 = substantial)

4. What are the most important opportunities presented by open science for your university? (First number is how many of the 12 respondents chose this item)

11 To gain access to the data resources necessary for research
9 To gain access to the software and tools necessary for research
9 To improve discovery processes
7 To gain access to the computational and storage infrastructure necessary for research
7 To increase industrial relationships and partnerships
6 To improve educational outcomes
5 To increase funding opportunities
5 To recruit students and postdocs
5 To increase recognition/rating of the university
4 To recruit faculty
3 Creating new curriculum to prepare students for careers in open science across different sectors
2 To improve pedagogical material and apply best practices for developing curricula

5. What are the most important challenges presented by open science for your university? (First number is how many of the 12 respondents chose this item)

10 To change the work culture of existing faculty and researchers
8 To reward open science work in tenure and promotion processes
6 To enhance library services for data curation and sustainability
5 To improve licensing, ownership, and other legal practices for open science
5 To develop technology infrastructure and staffing for open science
4 To create pedagogical material for open science curricula
3 To recruit faculty with experience in open science
3 Providing new teaching programs and training in open science
3 To develop a workforce for sustaining access to data and software
2 To recruit and retain non-tenure track faculty and staff

6. What is an open science success story that you find compelling?

The respondents mentioned:

- Cyberinfrastructure to support data sharing and collaboration, such as CyVerse (formerly iPlant) in the life sciences; the Southern California Earthquake Center (SCEC) initially funded as an NSF STC but with ongoing collaboration among 22 core institutions studying impacts of earthquakes on California; the INSPIRE platform in high energy physics which facilitates gatherings of scientists to review data, discuss new or expanded findings and then collaborate on publication; or at one institution a medical electronic data warehouse, with over six million de-identified patient records available for research across the university.
- Inclusion of the public and schools in science around open data and platforms, leading for example to the discovery of supernovae by elementary school children ^13^.
- The combination of the private sector with federal investments leading to new landscapes for knowledge creation, as in genomics.
- A European Union-like model for funding open collaborative spaces integrating people, publications, data, tools in a seamless manner, through a small tax on grants that then allows open shared data to be hosted for free and restricted access data to be hosted with a fee.

7. How can the outcomes of this workshop help your relationship with your stakeholders (such as your board of trustees or alumni)?

The respondents mentioned:

- Develop an authoritative report on the value of open science for the university stakeholders (both internal and external), including key points on current trends showing that this is a critical shift in higher education; exemplar outcomes showing the importance for discovery, current adoption issues, and recommendations/narratives/specific implementation strategies for institutions wishing to embrace open science/research.
- Develop principles for supporting open science to help universities attract and grow students and young faculty as well as industry and government partners.
- Connect open science to the topic of reproducibility and rigor/transparency in research ^a^
- While internal stakeholders (faculty, postdoctoral researchers, students) were generally seen as important, for universities dealing with classified and sensitive information, there is a responsibility and opportunity to educate their boards about data and open science.

8. What other related issues are important that we should discuss at the workshop?

The respondents mentioned:

- The federal government has a diversity of requirements for data sharing, and a diversity of financial models (not just the commonly encountered unfunded mandate.)
- The international landscape and international collaboration threats.
- Is there a way to articulate both intellectual and monetary value of open science to a university?
- Reward and recognition structures for those working primarily on data, algorithms and computational models. Tenure and promotion policies and their implementation.
- Who has responsibility to curate the data (not just to store it)? For example, who develops the metadata that allows one to best use the data?

9. If you want to elaborate on any of the answers above, or provide any other inputs, please do so here

The respondents mentioned:

- At the moment, there is stronger interest than there is investment or implementation at our university. This is primarily because much of the infrastructure and appropriate research practice needs to be developed, and this path is not completely understood.
- Open science works best when communities of researchers develop standards for data sharing. Simply making data available is insufficient. There needs to be national leadership to ensure common data standards and shared libraries.

## 3 Workshop agenda

The workshop began with an introductory dinner, where attendees had the opportunity to meet each other, learn a little about each other’s backgrounds, and talk about what they wanted to get out of the workshop. The next morning began with brief remarks intended to set the stage for the workshop: by Ed Seidel on behalf of the university leaders; by Dan Katz on behalf of the organizers; and by Stuart Buck and Rajiv Ramnath (remotely) on behalf of the funders. The group then discussed how to divide up the topics, deciding to organize around

- Credit and attribution
- Communities
- Education
- Outreach and engagement
- Preservation and reproducibility, and
- Technologies

while recognizing that there would be overlaps and cross-cutting issues.

## 4 Discussion topics and principles

Most of the remaining time at the workshop was spent with the participants divided into groups discussing the topics mentioned above. The groups generally discussed the assigned topic, and typically identified a small number of principles associated with the topic. Late in the day, each group presented its results to the full workshop and received feedback. During these discussions, at least three cross-cutting topics were identified: libraries, career paths, and campus information technology organizations & data repositories; see Section 5. The workshop attendees also discussed how to write up the workshop and future steps.

### 4.1 Credit and Attribution

Researchers respond to a variety of incentives in their daily activities. Many of these revolve around personal job security, hiring, promotion, and tenure. Credit and attribution form a core part of this, since the labor of evaluative job panels is often delegated to publication venues. This creates restrictions on both the types of practice that researchers will undertake and the forms of material that they are willing to publish. Without incentive structures that measure and value open scholarship, we are unlikely to see a large-scale transition.

The current set of assessment, credit, and attribution systems in the academy do not respect a diverse range of outputs, products, and activities. Instead, they coerce innovative work into known media forms in order to be congruent with assessors’ expectations. As a result, those working on digital and software outputs are often disadvantaged in the academic credit ecosystem. Those who collaborate on projects also fare badly by current standards, with poor recognition of non-authorial contributors. Those producing non-traditional research outputs would gain by changes to university assessment and credit/attribution procedures, since much contemporary scholarship and research now rests upon software and data outputs, which must be properly attributed. Proper credit for both traditional and non-traditional works also depends on avoidance of plagiarism.

However, it is also apparent that different types of “credit” exist, and that this is not a homogeneous term. Credit and attribution may work differently for those who do not seek a traditional tenure-track road in the academy. University leadership is often wary of intervening in decisions about assessment as they do not wish to be seen to encroach upon academic freedom. We also found that there was an increasing sense of a need to hire new types of faculty/staff and to actively develop criteria for their assessment in order to maintain a global open research infrastructure.

Stakeholders in the credit and attribution space are many and range from: early-career, mid-career, late-stage researchers, faculty who sit on committees, university administrators, funders, publishers, and metric providers. Late-stage and tenured faculty have more chance to experiment in this domain since the consequences at their appraisals are far less serious than for those without secure employment.

Economic imbalances between different stakeholders are also present in this space. Metric providers and publishers, for instance, derive economic benefit from becoming evaluative frames. Apart from being poor scholarly practice, such evaluation of containers (presses, journals) restricts the type of researcher outputs that are incentivized. We need to move away from the journal impact factor or container name as a proxy for research evaluation as also suggested by previous initiatives, for example the San Francisco Declaration on Research Assessment (DORA ^b^).

***Principles***. We defined the following **principles for credit and attribution**:

A1 Stakeholders (funders, universities, and researchers) should incentivize credit and attribution for a diverse range of research products and activities, such as research software, dissemination, infrastructure, data products and repositories.
A2 Research should be assessed on its own merits, not based on its appearance in exclusive publication venues. This could be through the use of article-level metrics or narrativized impact measures. Journal impact factors should not be used to evaluate researchers.
A3 Institutions that comparatively measure attention scores should evaluate attention measures with care, since such altmetrics may not correlate with measures of quality.
A4 Institutions should provide appropriate career paths for staff working on open research and maintain the institutional infrastructure required for open research, including recognizing and valuing new, emergent forms of digital outcomes, such as software and data creation, curation and preservation, that are crucial to open research endeavors. These pathways may require rethinking existing classifications and assessments of tenure-track, non-tenure-track, and staff categories of university participants, and funder support for personnel in these categories.

### 4.2 Open Scholarship Communities

Communities are the fabric of open research, and serve as the basis for development and sharing of best practices, building effective open source tools, and engaging with researchers newly interested in practicing open research. Effective communities often emerge from bottom-up interactions, and can serve as a support network for individual open researchers. These communities can consist of virtual clusters of like-minded individuals; they can include scholars, librarians, developers and technical staff or open research advocates at all levels of experience and with different backgrounds; the communities themselves can be short-lived and focused on a specific issue, tool, or approach, or they can have more long-term goals and aspirations. A key defining feature of these groups is that the principles of open scholarship permeate their practice, meaning they aim to be inherently inclusive, and aim to open up the process of scholarly exploration to the widest possible audience.

***Examples of success***. In the meeting, we discussed ^c^ different examples of (successful) Open Scholarship Communities, and ways in which these have been developed. To begin with, although successful Open Scholarship Communities (OSCs) collaborate on infrastructure, they can still compete on science: this extensive collaboration does not mean that the science is de-scoped, or there is less competition between researchers. For a successful collaboration, the perceived value of participating has to be greater than fear of consequences. In other words, participation to Open Scholarship Communities should increase value (“I don’t have to reinvent this”) or decrease fear (“I can use a standard someone else invented!”). This is not an all-or-nothing step: it is important to praise incremental steps and make it easy (if not automatic) to continue ‘open’ behaviors.

For any of this to happen, and to make sure that the barriers to participation are not too great, systems and tools should emphasize the lowering of barriers to entry, resource efficiency and productivity: much can be gained in the tool/middleware layer. This also means that those creating those systems and tools get credit for it. We propose a new metric where work on infrastructure development is valued. One idea would be to rate researchers on a ‘FISH’ scale: Funding, Infrastructure, Science, and H-Index: four dimensions that validate orthogonal contributions to scientific progress. It is necessary that the credit and attribution system is in place to provide examples of ‘infrastructure leaders’ (akin to research leaders) to help overcome the notion that researchers who work on infrastructure and tool development suffer with respect to funding, promotion/tenure and such. In fact, we know of several examples (including some of the workshop participants) of researchers whose careers benefited strongly from being involved with open infrastructure, tooling, and community activities.

***Recommendations***. After collecting a series of narratives on effective and intentional approaches to creating, growing, and nurturing such communities, we recommended the following actions for the different stakeholders to support the formation of adaptive and organic, bottom-up, distributed and open research communities:

For institutions, it is important, first of all, to provide the physical space and/or administrative support for community interactions. Since these practices are often not ingrained in the current research culture, institutions can support open scholarship by recognizing the need for explicit training in principles and practices of open research. This can and should include exploring what ‘design by a community’ looks like in areas where it’s not traditional, (e.g., mechanical engineering) and actively support changing views of what constitutes excellence in a discipline. Becoming more open is not a single step, but a process: it is imperative to reward incremental steps and provide incentives for engaging in different aspects of open scholarship at different levels. Engagement is more likely to occur if all steps are recognized as progress (some scholars will be happy to share their code, but not their data, or vice versa) and it’s easy to continue down a ‘sharing trajectory’ with incrementally greater levels and forms of openness.

For funders, it is important to recognize how ‘disciplinary shackles’ can hinder adoption of open scholarship practice. Development of common software, workflows and other community resources may not be respected as part of disciplinary work, but funders recognizing these non-traditional outputs can effect a culture change. A key component of openness is a focus on collaboration over competition: funders can contribute to making this happen by awarding grants to interdisciplinary and team efforts next to or instead of individual competitive efforts. Inclusivity is a defining feature of open scholarship, as well as extensibility and reproducibility. The goal is not solely to further individual rewards but to facilitate involvement of others: this means looking beyond ‘lock-in economics’ where the winner takes all, and exploring other reward systems. As with research institutions, funders should not adopt an exclusive definition of open scholarship, but reward incremental steps, by providing incentives for different aspects of open scholarship.

Publishers and (research) platforms (data repositories, standards bodies and such) can support the trend towards openness in various ways. First, they can build the process of openness into the platform interface, by making openness the easy option, enabling open scholarship training materials into the platform, and building social networks and sharing opportunities into the fabric of the user interaction. Platforms can lower the entry barrier towards sharing practices by helping to build and define communities (e.g., similar to “My Facebook friends” you can have “My Jupyter Friends”). When platforms support the creation of communities around specific tools and practices, this helps build norms and codes of conduct into these platforms endemically. Community development can be further enhanced by supporting the development of platform specialists inside institutions (e.g., “JupyterHub guru on campus”) and supporting “pop-up open scholarship communities” around specific tools and practices (e.g., “open data hackathons”).

As a fourth and last stakeholder, community organizers can build openness into governance by recognizing the value of simple narratives for attracting people into community participation. This means identifying and funding ‘culture changers’: people who are tasked with changing, e.g., data dissemination processes/practices, and people who bring a culture and practice of open scholarship into the community and are happy to share their knowledge, toolset, and experience with community members. The community can and should reward incremental steps towards openness by community members, to easily allow new members to join. To ensure that diversity in background, culture and experience is acknowledged and maintained, communities should establish and maintain a code of conduct and set of expectations regarding community interactions.

***Principles***. We defined the following **principles for open scholarship communities**:

C1 Scholarly communities are both the target and the product of open scholarship and open research. Therefore, the fostering of communities is a key driver for open research.
C2 These communities can take many forms. They may, for example, coalesce around tools, practices, shared interests, shared data or software, or shared hashtags. They can be short- or long-lived, explicitly funded, or emerge organically from shared interests among the participants. All of these variations are valuable components in the open research social ecosystem, and they must be supported as such by multiple resources, including travel funds, virtual networks, compensations for networking events, etc.
C3 Community activities often serve to evaluate, encourage, and improve the use of tools, software, platforms, and data that are critical to open scholarship. All stakeholders must take steps to encourage these communities to develop, such as supporting common standards (and rewarding those who work on them), and funding projects that form a “connective tissue” between different communities. They should also actively encourage sharing practices for tools, and people across communities.

### 4.3 Outreach and Engagement with the Public

The practice of scholarship has a natural tendency to result in insular communities uniquely driven to produce new knowledge within narrow disciplinary bounds. The communication of research and research findings through traditional modes of journalism, such as through newspapers and television, is often limited to only high-profile work with broad public interest. At the same time, the communication of the rest of research is a critical component of having an informed public ^14^. Further, since much of the funding for research and scholarship is derived from public money – as state or federal grants, tax funds, or student financial aid – the research community must find new and ever evolving ways to communicate with a diverse set of stakeholders, each of whom plays a role in ensuring that the societal and technological progress of our world is supported. This aligns well with the intent of the land-grant university under the Morrill Acts, which help define the missions of many higher education institutions and include a focus on outreach for an educated populace. On this basis, we proposed a set of guiding principles (see below) for outreach and engagement with the public. First of all, outreach and engagement, for public access to and understanding of research outcomes, depend on the audience and may encompass products, process, and dissemination. It is important to note that universities and researchers share a mutual interest in interactions with diverse audiences: therefore, outreach must be an institutionally valued, recognized, and supported component of university “service.” A key aspect of all of this is that access to products (research) and practitioners of research (scholars) builds trust in the products and process of research (scholarship).

Operating within a changing landscape of scientific reporting, researchers often find themselves in a position of needing to fill communication gaps through outreach and engagement with stakeholders. These stakeholders are diverse and may include members of the public, the media, policymakers, educators, science enthusiasts, industry, students, other researchers, and university administration. Each of these diverse groups of people represent different and sometimes competing interests. Accordingly, the message disseminated must be crafted to match each audience and their needs. This could range from the raw data generated during an experiment to a broad explanation of the scientific process aimed at improving general scientific literacy. In most if not all cases, it is not appropriate to assume that by making our work available through open access journals we are doing enough to make our work accessible to the public. For our work to be accessible, it must be both available and comprehensible by the general public. Within this line of thinking, some labs and departments have implemented public-engagement policies aimed at improving public understanding of their research or field (e.g., Harvard University Department of Astronomy Public Outreach Project ^d^).

The ability to communicate on such a variety of topics to such a diverse set of stakeholders is generally not a part of a researcher’s training. This means that institutions must invest in outreach and engagement techniques and support their researcher’s efforts in building this skill set. Success in these efforts can potentially be evaluated through assessment of the reach of such communications. Alternative metrics such as social-media influence or publications in traditional media will help university communication offices track whether efforts are having the desired effect.

***Principles***. We defined the following **principles for outreach and engagement with the public**:

O1 Outreach and engagement, for public access to and understanding of research outcomes, depend on the audience and may encompass products, processes, and dissemination.
O2 Universities and researchers share a mutual interest in interactions with diverse audiences.
O3 Institutions should value, recognize, and support researchers who participate in outreach and engagement activities, crediting them as components of "service" to the institution.
O4 Access to researchers (scholars) builds trust in the products and processes of research (scholarship).

### 4.4 Education

Open scholarship can have an impact on improving education. In order to prepare students for emerging careers, accelerate discovery and reduce redundancy, incorporating open scholarship can bring about more opportunities and is critical for the survival of universities. The idea of Open Educational Resources goes back about 30 years, when advocates of “open content” proposed that principles of Full Option Science System (FOSS) could be applied to educational materials. (The term Open Educational Resources, OER, was coined at the 2002 UNESCO Forum.) Recurring topics in OER are reducing cost (for students), and increasing access.

To implement these ideas, we discussed examples that were relevant to these suggestions and were successfully adopted. As most of the workshop participants are University faculty, many of our recommendations were geared toward undergraduate level education. However, the sooner we can implement the idea of open scholarship, the greater the impact for future generations, and as university faculty, we should work with K-12 teachers to prepare and train them for implementation of open scholarship concepts. To do this at the university level, we first propose to embed open scholarship practices in the current curriculum for each major, as has successfully been demonstrated at UC Berkeley, aspects of which have been summarized in 15. Second, we propose to identify key faculty leaders in open science and incentivize engagement of open science with innovative methods such as funding course buy-outs. Third, we propose to develop low-barrier training and communities for students, staff, and faculty to engage in open scholarship and its benefit. A carpentry website ^e^ that teaches foundational coding and data science worldwide perfectly embodies this concept. Last, we propose to introduce the concepts to K-12 teachers to prepare and introduce the concepts as early as possible.

Many opportunities are available to drive universities towards open scholarship. Some, such as re-evaluating the university’s educational role, are more challenging, while others may pose lower barriers, such as positioning land-grant universities to provide a more contemporary role for education by merging research and education. A critical component for many students today is the gap between education and research. Most undergraduate students never have the opportunity to be exposed to research, which is critical for creativity and accelerating discovery, and can increase the relevancy of education.

Revamping curricula comes with obvious challenges, such as changes that might involve university structure, training instructions, and changes in materials. However, due to the fast pace and constant change in research, textbooks in classrooms are becoming less useful, particularly in upper-level undergraduate courses. We have a window of opportunity, due to outdated textbooks, to introduce concepts of open scholarship. Open scholarship can help accelerate discovery by not depending on old literature, reducing redundancy, performing higher quality and more efficient research, and lowering barriers to collaboration by building resources for broader communities. Metrics are also needed that can be used to define the success of open scholarship for education to be incorporated in classrooms; in order to drive change, there needs to be a way to define the success of open scholarship in the current examples.

One example of open scholarship at the University of Arizona involves a researcher in drug discovery and basic sciences, May Khanna, who has created a course named “From Chemistry to Cure,” incorporating concepts of open scholarship. The course will begin with virtual docking of targets chosen by the students using cloud computing as has previously been done ^f^. The students will continue the process of virtual drug discovery through the course, uploading their results on a live blog. The students will then complete the course by pitching their concepts to business students and results from pitches will continuously be shared. The course will include live student quizzes with such software as Poll Everywhere and apps that allow for cloud sharing of information. The idea of open scholarship in the course allows it to be integrated with other universities throughout the world, which will be done as the course matures in future years. This open scholarship course touches on several critical points that were discussed in the workshop: it merges research with education; it utilizes cloud computing for increased computing power through platforms like Google or Amazon and thus is not limited by local computers; it merges research goals with education using an open forum to teach students to be creative, open, and to accelerate discovery; and it shares materials with an international open forum, which breaks down the barrier between research and education. The course is completely driven by the students, which gives the students greater responsibility.

***Principles***. We defined the following **principles for education**:

E1 Every student should be guided to understand and learn how to access and use data and software to be well prepared for diverse, modern career paths.
E2 Through open research training, universities should play an active role in increasing research by enabling evidence-based decisions, accelerating discovery, and extending impact to broader communities.
E3 Universities should encourage their faculty to engage in open educational practices, including creating and assigning open educational resources, and reflecting open culture in their courses.

### 4.5 Preservation and Reproducibility

Reproducibility depends on transparently documenting and sharing all data products, protocols, and computational algorithms (with source code) used in the research. While advocates of open, reproducible scholarly research believe that it should be the norm—coupled with data sharing, reusability, and sustainability more generally—individual and institutional barriers hold back wide adoption of such practices and workflows. Currently, while some individuals and research groups feel strongly about openly sharing all research products (including data, written output, and software) and working in a reproducible way, they are largely motivated by personal beliefs. (Some scholarly communities have developed cultures with some aspects of openness, e.g., in physics the sharing of preprints via *arXiv* is the norm; but this is not widespread.) Thus, incentives at both institutional and wider community levels are needed to initiate change.

Community leaders can and should make positive arguments for sharing and reuse of digital artifacts of research. These arguments could be more successful than negative ones around lack of reproducibility (the “crisis narrative”). Although arguments for open data and software often focus on increased citations, we can also argue for the greater overall impact and opening up of new, collaborative avenues of research. Similarly, while funders and publishers may support reproducible research in theory, in practice they currently provide few incentives. Funders could precipitate change by making reproducibility concerns part of the merit-review criteria; they could require compliance with data management plans for any future support, and extend such plans to consider software explicitly (e.g., “Data and Software Management Plans”). Publishers can award badges to articles that present open and reproducible workflows, for example, as is the case in the *ACM Transactions on Mathematical Software* ^16^, or could go even further by encouraging editorial boards and reviewers to only consider submissions with such workflows. For example: the *American Journal of Political Science* contracts a third-party to verify that author-provided files are sufficient to reproduce the results in the paper. The Odum Institute ^g^, in this case, carries out reproducibility checks of accepted papers, and authors submit any required additional information before publication.

Reproducible workflows require ensuring access to open and reusable data as well as open, reusable, and sustainable software. Data should be preserved following established community standards, such as the FAIR principles – data should be Findable, Accessible, Interoperable, and Reusable ^17^. An analogue to the FAIR principles for data does not exist for software, although recommendations have been made for the specific case of applying fair-use principles to allow preserving software for posterity ^18^. However, for the purposes of reproducibility, research software needs to be made open source at the time of publication of the research results. Perhaps more importantly, it should be written from the outset with sharing and reuse in mind (ideally under an open development model). A new initiative, *The Journal of Open Source Software*, provides peer review on open code and promotes good practices for preservation (an OSI-approved license is enforced and software must be persistently archived before the paper is published). Leaders at all levels should also encourage scholars to appropriately cite data and software when used for a study, similar to citing literature articles ^19, 20^, to help standardize this behavior.

In addition to communicating the importance of preservation and reproducibility, training in skills for open and reproducible workflows needs to be emphasized, noting that skills for reproducibility are not the same as those for working openly in general. This could be another new role for libraries, but some work may be discipline-specific, and training by faculty should also be recognized as a service contribution. One good example of this is C. Titus Brown’s position at University of California Davis, which involves lower “traditional” teaching loads but more service in the form of computational and data science training for biologists and bioinformaticians.

***Principles***. We defined the following **principles for preservation and reproducibility**:

P1 The scholarly publication and communication ecosystem should support open and reproducible research, and enable credit for these efforts. Universities should encourage these initiatives by creating incentives (e.g., promotion and tenure categories, service recognition) for such activities.
P2 Research funders should support open and reproducible research by making reproducibility part of their merit review criteria. They should also create new scholarly communication venues or support open scholarship efforts, and encourage, require, and reward reproducible research efforts.
P3 Incentives that promote the public sharing and distribution of scholarly knowledge for open/transparent/reproducible research practices must be put in place. Publishers must require, when appropriate, submissions that provide open and reproducible workflows, and embed this requirement in their own workflows.
P4 Universities should recognize the activities of faculty to educate and train researchers on open and reproducible research skills. Global and national bodies (e.g., National Academies) should promote this recognition across universities.

### 4.6 Technologies

Enabling open, collaborative scholarship that engages students, researchers and the public requires reducing the barriers to entry for both generating and distributing knowledge “products.” Academic endeavors—in both research and education—reap most benefit from adopting open source software in all technologies they adopt. Utilizing closed-source software creates a number of impediments to effective research, including reducing verifiability of the research products, reducing opportunities for synergistic collaborations, and imposing barriers to entry for reproducibility and dissemination of knowledge. In addition to these pragmatic considerations that favor open source software, we also identify that open source technologies provide intrinsic value to the entire research process that extends beyond a monetary value proposition. We therefore not only recognize that open source technologies should be preferred to accelerate open scholarship, but that researchers should be appropriately acknowledged and rewarded for participating in the ecosystem of open source and open data for scholarship.

In support of embedding open source technologies for open scholarship, we propose that institutions prioritize the identification and (ad hoc) endorsement of capabilities that meet several criteria. Firstly, the tools should be both interoperable and, to the extent possible, self-documenting. This provides the ability for individuals to communicate between different pieces of software and technical infrastructure, ensuring they are able to transport their work as the situation requires. An example of this is in data formats and storage methods, as well as in the ability of in-memory transfers between software libraries, or in the execution of virtual machines and containers on different cloud providers.

The second characteristic of tools that we highlight is that they should be selected to reflect a wide-ranging set of evaluating criteria. Rather than determining “winners,” these tools should be drawn from a diverse, evolving set of possibilities. Entrenching a single technological choice may serve to unduly influence future research studies and restrict growth of scholarly technologies.

We also recognize that frequently the ability of researchers to utilize cutting-edge open source and open scholarship infrastructure can be subject to the bottleneck of their own technical skills. This imposes a technical barrier to entry that we believe will detract from the utilization of open scholarship tools and software. To mitigate this, we propose that the University of Tomorrow provide foundational infrastructure, in the form of both technical resources (deployments, hardware, “glue” software) and human resources (support staff, contributing members of the open source community) to ensure that these tools and opportunities are made available widely across the university, coupled with learning opportunities that allow sharing of best practices.

***Principles***. We defined the following **principles for technologies**:

T1 Open source technologies, tools and platforms provide intrinsic value to researchers and educators and are an effective way of accelerating open scholarship. Academic institutions should favor and encourage open source solutions as much as possible.
T2 A diverse and interoperable set of tools for open research should be known, shared, and clearly documented.
T3 Institutions should provide and support foundational open scholarship infrastructure (technological and human) for all members of campus.
T4 Institutions should recognize the contributions made by all members of campus to open scholarship infrastructure.

## 5 Cross-cutting topics

Three cross-cutting topics were identified in multiple group discussions: libraries, career paths, and campus information technology organizations & data repositories, though there are likely other parts of the university that should be involved in future discussions, such as policy and research offices.

### 5.1 Libraries

Every research university has a library whose mission is to support research and education by collecting, organizing, managing, preserving, and ensuring long-term access to the products of research and the scholarly record for every discipline. Librarians and other library staff provide expert support to students and researchers at every career stage, universally and democratically. In many ways, libraries are the original core research facility, inseparable from the university itself, evolving alongside technological advances and other changes in research and educational models and methods. As we consider the future of research universities, open scholarship, and data-driven research, the unique role that the library plays in the university needs to be re-envisioned and perhaps broadened.

Many of the challenges and opportunities facing research universities in embracing open scholarship and data-driven research revolve around “scholarly content” – recorded knowledge in books and journals but also in software and models, datasets and databases, visualizations and vast digital libraries. Libraries are already adapting to include these new forms of scholarship in their traditional functions of collecting, organizing, describing, preserving, and providing ongoing access. But digital content is different than print and other analog formats, providing greater challenges and opportunities at the intersection of research and content, on the production and consumption sides.

Specific ways in which libraries support open scholarship and data-driven research include the following.

Libraries play a key role in helping institutions and individuals document their impact on knowledge creation. New forms of scholarship and research (e.g., scientific software or complex data creation) require new forms of credit and attribution to measure their impact in ways that can be aligned and integrated with traditional credit mechanisms: citation and attribution. The current scholarly record system was designed for authorship and is maintained by libraries and information companies (e.g., Web of Science), and libraries are collaborating with researchers to develop new citation standards and methods, promote new disciplinary norms, and create new tools and databases that interweave new and traditional scholarship. Libraries train students in citation practices and bibliographic tools, and collect the data that provide evidence of impact, including alternative metrics to traditional citation, such as ‘altmetrics,’ documenting both institutional and individual impact ^21^.
Libraries are natural homes for interdisciplinary research communities to form and collaborate. They provide central, neutral, and welcoming facilities, often with shared equipment and other research tools that are expensive and inefficient to duplicate in multiple departments (e.g., 3D printers, visualization tools, specialized software). Importantly, library spaces bring together researchers and students from across the university who are then exposed to each other’s research methods ^22^. This is analogous to “browsing the stacks” of 20th Century libraries but with people and research as the objects of serendipitous discovery.
The fact that libraries are naturally omnidisciplinary allows them to organize quickly and fluidly around new research constellations without the need to form new “disciplines,” with consequent norms for publication, curricula, and excellence. They can attract and often hire academic staff to run new research areas (e.g., data science or spatial science researchers) and provide them with avenues for recognition and advancement, if not tenure.
Libraries are logical stewards of and repositories for open scholarship technologies and data ^23^. They provide expert support to all researchers and students for particular tools (e.g., bibliographic management tools, GIS software systems, 3D printers, text mining or bioinformatics) and practices (e.g., data curation, software publishing and archiving). They have the technical skills to catalog and organize the new products of open scholarship (e.g., data and software), individually and collectively via national consortia and programs ^24^. For open scholarship to become the default, it will take coordinated international efforts to manage the tools and products of research, and libraries are well-positioned to expand current networks to meet that need.
A core value of libraries is knowledge sharing – that everyone should have free and frictionless access to all knowledge for all time, whoever and wherever they are. Because of that value, libraries are strong advocates of open scholarship – open access, open data, open educational resources, open methodologies and research reproducibility – and promote them to their own communities and to the public at large. As an illustration of this advocate role, libraries are providing leadership in the development and support of open scholarship policies. Library professionals contribute expertise on open scholarship issues to government and university leaders as policies are being written. As new policies emerge and evolve, library staff are then often central to campus initiatives focused on meeting new policy requirements.
Libraries can provide skills training at the point of need and in flexible ways, unlike traditional academic departments ^25^. These training offerings can be credited (e.g., a semester-long seminar) or uncredited (e.g., a two-week boot camp or a single class) to allow for different incentives and rewards to students. The central locality of these training sessions exposes them to other students and researchers, providing a novel form of marketing and outreach.
The library’s mission to acquire, organize, and ensure permanent access to recorded knowledge means that it has already begun serious exploration of methods for digital preservation and research reproducibility. Since the 1990s, libraries have been developing best practices for describing and preserving many types of digital research products, e.g., CAD models, software libraries, images of many formats, audio and video. Libraries are key partners to research-generating agencies for ensuring long-term access to digital content, and to operationalizing support for that content over decades and longer ^26^.

The main challenge that libraries face in building support for open scholarship and data-driven research is the same one facing their parent institutions: the need to balance supporting traditional modes and methods of conducting research and education, still critical for many functions and people across these institutions, with investment in new types of support for the new institutional goals ^27, 28^. Transitions can take decades and straddling two worlds is complex and expensive. To find the right balance of investment and pace of change, clear guidance is needed from both university administration and from the researchers and students themselves. Libraries already regularly build collaborations across campus units (e.g., with campus central IT/computing units and research administration offices) to ensure that research support services are coupled and coordinated. Such collaborations will continue to grow in importance.

The transition toward open scholarship is occurring unevenly across institutions and disciplines, so we find incoherent systems of practice today. Beginning with disciplines that are further along in the transition, defining global strategies for success, building collaborations, and focusing investment to implement those strategies, are good places to begin.

### 5.2 Career paths for researcher-developers

New models for supporting career paths that involve research, development, and campus service are emerging. Today, these positions can be considered along multiple axes, including professional status (postdoctoral researcher, staff, traditional tenure-track faculty, teaching faculty, research faculty, and faculty of practice), length of position (short term, long term, permanent, tenured), funding (soft/grant funded, institutionally funded), and organization (in one or more academic department(s), in an IT organization, in a center or institute, the library, or a combination). This is a large and diverse space of possibilities, and it is both possible and likely that universities may create new career paths with little overlap in their models. Hence, there is value in experimentation and in the insights gained from these experiments.

A number of common elements appear, including:

- The criteria used in hiring, evaluation, and career advancement.
- The mix of job responsibilities, including teaching, research, service, and community engagement.
- The positioning of software development and maintenance relative to knowledge creation, preservation, and dissemination. For example, it might be part of responsibilities in a traditional classification, or part of a new one.
- The remuneration models and their relationship to those of other career paths at the same institution, and professionals with similar skills making a career in industry.
- Transition models into and from this career path, both within and across institutions.

In one example of such a model, from the University of Washington (UW), the Provost supported half faculty lines to help recruit data-oriented and software-oriented faculty across campus, and to help give them a community. In return, these faculty engage in teaching courses relevant campus-wide. Their tenure case is still owned by the home department. UW also explored chaired professorships in various fields funded through a Washington Research Foundation grant. These titles help reinforce community engagement and leadership among faculty around campus.

Perhaps more uniquely, the UW eScience Institute has developed strong career paths for data scientists, typically with PhDs in STEM fields and strong software development expertise. These are very different roles than typical Research Software Engineers, which we find tend to be interpreted as service roles rather than senior researcher roles. The model for these data scientists is:

- PhD in domain science, with significant experience in data and/or software.
- 50% work on Institute projects and initiatives, and 50% on their own research. (Typically 100% involves collaboration with others around campus.)
- Autonomy to choose which initiatives they work on.
- PI status for non-junior roles.
- Affiliate faculty roles, where that makes sense.
- If they buy out their time by being part of a grant, they get some of the salary savings returned as research/travel/student budget.
- They are seen as "faculty peers." For example, sometimes working titles have significant influence: "Director of Research in the Physical Sciences" rather than just "Data Scientist."
- There is a community of these Data Scientists – shared governance, shared space, shared initiatives, social events. This avoids them disappearing into other peoples’ labs, where they would risk losing their autonomy and respected status.

This model appears to be replicable, perhaps by exploiting attrition in IT departments and libraries to build up a community of Data Scientists.

### 5.3 Campus information technology organizations & data repositories

Universities collect massive amounts of data for research, teaching, learning, service, outreach, and strategic management. These data collections expose universities to new risks and create responsibilities that may converge and diverge in unexpected ways. Drawing on recent work ^29^, this short section examines university concerns for research and “grey” data gathered for administrative and operational purposes.

By collecting data, institutions assume responsibility for managing those data in the short and long term. “Stewardship” is an overarching term that encompasses sustainability, curation, access, and preservation. Although “stewardship” is used in nuanced ways in the scientific, library, archival, and policy communities, it reflects a commitment to managing data in ways that they remain findable, accessible, and useful. For some kinds of data, stewardship requires indefinite preservation; for others, regular cycles of record disposal are needed. Modern data collections are dynamic, thus traditional archival approaches to sustaining access to static resources are unlikely to suffice. In an “age of algorithms” where datasets are in constant flux and can be disaggregated and re-aggregated continuously for multiple analytical purposes, new approaches are sorely needed ^30^.

Universities have broad responsibilities for stewarding the data they collect, acquire, and hold. Despite the diffuse responsibility borne by institutions, some individual persons, offices, committees, or other entities must take specific actions, make investments, and manage the daily operations of data stewardship. Determining which entities have which responsibilities, based on what criteria and policies, is the process of governance. The University of California was among the first to address these processes in U.S. higher education, explicitly acknowledging the “distributed nature of information stewardship at UC, where responsibility for privacy and information security resides at every level” ^31^. Universities are taking many approaches to governance, ranging from appointing “data czars” to assigning offices or committees to formulate generalized policies, agreements, and governance mechanisms.

Whereas universities are generally held responsible by funding agencies for maintaining data, the responsibility for disposition and stewardship usually falls to the researchers who collected those data. They have vested interests in exploiting and protecting these data and they know the most about the data’s content and context. Local knowledge is essential to data management, given the vast array of data types, domain expertise, policies, and practices. However, the benefits of local control must be balanced with expertise and continuity. In units with external funding, graduate students and post-doctoral fellows conduct most of the data collection and perform most of the management tasks. They also write software code, scripts, and algorithms to analyze those data. Rarely are these domain experts also experts in data management or software engineering. Essential research tasks are being performed by short-term employees who are replaced every few years as students graduate, fellowships end, and grant projects are completed ^32^.

In many academic domains, authors and investigators are responsible for releasing datasets associated with publications. Finding and funding access to their data for some specified number of years after the granting period is a looming challenge. Where data archives exist, deposit is usually the preferred solution, whether organized by discipline, data type, or institution, as these entities tend to have long-term commitments and staff responsible for curation. Archiving of digital research data has been under way for at least fifty years by entities such as the World Data Systems ^33^, Harvard’s Institute for Quantitative Social Sciences and Dataverse ^34^, and ICPSR ^35^. Funding agencies vary considerably in support for sustaining access to findings. Some provide data archives, others require universities to maintain their own data archives as a condition of receiving grants, and yet others are agnostic on the disposition of datasets, as long as they remain accessible. Sustaining access to public archives is itself a challenge, as many of these are funded by research grants that expire on a cyclical basis.

## Conclusions

The workshop “Imagining Tomorrow’s University” was a unique gathering of early career faculty and university leaders, united in their efforts to understand the implications of open scholarship on future universities. They worked together to derive the 22 principles listed above based on their personal goals as well as their mutual interest in seeing that universities take best advantage of opportunities brought about by current changes in scholarship and society, including increased digital products, increased sharing and transparency, public skepticism in authority, and the perceived reproducibility crisis.

Other groups are also working in this area. Since the workshop, the National Academies has written a report on how to accelerate the movement towards open scholarship ^36^ and the AAU-APLU Public Access Working Group released a report describing principles and recommendations for agencies and institutions to consider in implementing infrastructure for sharing research data ^37^.

Activities that follow on from workshop results take one of three forms. First, since the workshop, a number of workshop authors have further developed and published their white papers ^38– 41^ to make their reflections and recommendations more concrete. These authors are also conducting efforts to implement these ideas, and to make changes in the university system. For example, one of the current authors (Erin McKiernan) recently collaborated on a project to analyze review, promotion, and tenure (RPT) documents from a representative sample of 120 universities in the U.S. and Canada to learn how the public dimensions of faculty work, including aspects of open research, are currently valued and rewarded in university evaluations ^42^.

Second, we propose to organize a follow-up workshop that focuses on how these principles could be implemented. This workshop could include 4–7 research institutions, some of which were represented at the workshop described in this report, and some of which would be new to the table. It would also include participants from a greater diversity of positions at these universities, including early career researchers, senior faculty, department chairs, deans, librarians, research data managers, as well as higher-level university leaders. This workshop would aim to work out the details by which a set of changes could be made, and conduct a trial to implement at least some of those changes at each of the participating institutions. The workshop would identify clear objectives and key results for each of these trials, as well as metrics by which they could be assessed.

Third, we believe that the outcomes of this workshop support and are connected with recent theoretical work on the position and future of open knowledge institutions. In particular, the recently published online monograph “Open Knowledge Institutions: Reinventing Universities” ^43^ advocates that universities “become Open Knowledge Institutions which institutionalise our world’s creative diversity in order to contribute to the stock of common knowledge.” The authors argue that such Open Knowledge Institutions “act as networks of knowledge, spanning common disciplinary boundaries and campus barriers in order to serve as agents for societal change.” The work proposes a theoretical change mechanism for knowledge institutions to become more open, which we believe can be a useful frame of thought, as well as a means of studying change in knowledge institutions towards greater openness. It is argued that the change from closed to an open (knowledge) institution happens in three stages:

(i) policy and intent signaling (where action is desired and expressed);
(ii) action and investment (where action is being taken); and
(iii) measurable outcomes (where the result of action is assessed).

To make sure that actions undertaken do indeed lead to greater openness, three aspects should be taken into account:

- Since one of the core goals of open institutions is inclusion, **Diversity** is essential to this change, to ensure participation from a broad group of stakeholders, including nontraditional/unfunded/formerly peripheral actors (including the general public and local/nonlocal parties interested in university efforts);
- To ensure a productive transition, extensive **Coordination** needs to be done between the (many) different groups involved, who all have different directives, backgrounds, requirements, and levels of knowledge and interest;
- To make sure that Coordination occurs and Diversity is achieved, **Communication** is needed, both to transmit knowledge to the diverse communities and to support and engage diverse groups in the extensive, multi-stakeholder dialogues that characterize open institutions.

Connecting these stages to these aspects of inclusion leads to a 3 x 3 table through which actions towards opening up knowledge institutions can be classified (e.g., see Table 1 in “Open Knowledge Institutions: Reinventing Universities” ^43^). As a final follow-up to our workshop, we propose to use this framework to position and track efforts proposed and undertaken at the research institutions that take this work forward. We have commenced conversations with the authors of the monograph and will invite them to participate in these follow-up steps, to further enhance our collective understanding of this pivotal transition happening in academia today, from ivory towers to open knowledge institutions.

## Data availability

No data is associated with this article.

### Appendix

Early Career Research Leaders

- Devin Berg (mechanical engineering), University of Wisconsin Stout
- Holly Bik (bioinformatics, genomics), University of California Riverside
- Carl Boettiger (theoretical ecology), University of California Berkeley
- C. Titus Brown (bioinformatics, genomics), University of California Davis
- Martin Eve (English literature), Birkbeck, University of London
- Brian Granger (physics, data science), Cal Poly State University San Luis Obispo
- Adina Howe (bioinformatics, genomics), Iowa State University
- Bill Howe (computer science), University of Washington
- May Khanna (structural biology, drug discovery), University of Arizona
- Matthew Mayernik (research data management and curation), National Center for Atmospheric Research
- Erin McKiernan (biophysics, physiology, neuroscience), National Autonomous University of Mexico
- Kyle Niemeyer (mechanical engineering; computational combustion and fluid dynamics), Oregon State University
- Matt Turk (astronomy, data science, information science), University of Illinois Urbana-Champaign

University Leaders

- Randy Burd, Associate Vice President for Research, Global Research Alliances, University of Arizona
- Jean-Lou Chameau, President, King Abdullah University of Science and Technology
- José-Marie Griffiths, President, Dakota State University
- Randolph W. Hall, Vice President, Research, University of Southern California
- Timothy L. Killeen, President, University of Illinois
- Sarah M. Nusser, Vice President for Research, Iowa State University
- Cybele Raver, Senior Vice Provost for Academic Analytics and Graduate Academic Affairs, New York University
- Daniel A. Reed, Vice-President for Research and Economic Development, University of Iowa, now Senior Vice President for Academic Affairs at the University of Utah
- Edward Seidel, Interim Vice President for Research, University of Illinois
- Jay Walsh, Vice President for Research, Northwestern University

Other Stakeholders

- Laura Noren (organizational sociology), New York University
- MacKenzie Smith (research library), University of California Davis
- Jeffrey Spies (research technology and methodology), Center for Open Science

Funding Organization Representatives

- Stuart Buck, Laura and John Arnold Foundation
- Josh Greenberg, Alfred P. Sloan Foundation
- Chris Mentzel, Gordon and Betty Moore Foundation
- Rajiv Ramnath, National Science Foundation (remote participation)
- Carol Shreffler, National Institutes of Health

Organizers

- Gabrielle Allen, University of Illinois at Urbana-Champaign
- Lorena Barba, George Washington University
- Christine L. Borgman, University of California Los Angeles
- Anita de Waard, Elsevier
- Daniel S. Katz, University of Illinois at Urbana-Champaign
- Nirav Merchant, University of Arizona
- John Van Horn, University of Southern California
- Edward Seidel, University of Illinois

Facilitators

- Stavros Michailidis, KnowInnovation
- Donnalyn Roxey, KnowInnovation

## Footnotes

^a^ https://web.archive.org/web/20181020160025/https://research.usc.edu/rigor-transparency-and-reproducibility/.

^b^ https://sfdora.org

^c^For the full set of notes, see https://web.archive.org/web/20170709184934/http://ivory.idyll.org/blog/2017-open-science-communities.html.

^d^ https://web.archive.org/web/20180808184837/https://astronomy.fas.harvard.edu/public-outreach-project.

^e^ https://carpentries.org/.

^f^ https://web.archive.org/web/20170912204801/https://cyclecomputing.com/improving-als-research-with-google-cloud-schrodinger-and-cycle-computing/.

^g^ http://odum.unc.edu/.
