# Supplementary material for: The principles of tomorrow's university
Source: F1000Res. 2018 Dec 11;7:1926. [Version 1] doi: 10.12688/f1000research.17425.1 (PMC6338243; doi:10.12688/f1000research.17425.1)
Supplement: Peer review history

# Peer review: Referee response for version 1

**DOI:** 10.5256/f1000research.19054.r42058

**Published:** 2019-01-14

**Authors:** Olivia Guest

**Affiliations:** 1 Department of Experimental Psychology, University College London (UCL), London, UK

This paper summarises the ongoing dialogue around open scholarship between ECRs (and others) and people in relatively more powerful positions within institutions based on the outcomes of a workshop, which included representatives from these two groups, held in March 2017 and in the USA. The points raised in the workshop and thus this paper are of value to the wider open scholarship community, to libraries, to institutions and funders and to society in general.

 In my opinion, this article needs some minor polishing only. My comments below attempt to help the authors achieve a more clear version of their paper.

 The Abstract and Summary could contain a sentence (near the top) that explicitly states what the article is about. I was a little lost without this. Something like "This article is about X, Y, and Z." — not unlike the first sentence of my review. I wasn't sure if the workshop mentioned what was the main focus of the article or if the authors were themselves present until I checked what was being said further along in the manuscript. I wondered if the current article was going to elaborate on their viewpoints or add to them, etc. Along the same vain, in the paragraph that begins “In March 2017”, in the Introduction section, I think it would help the reader to know which of the attendees are authors? I know they are listed in the appendix, but I suppose a reader would have to manually check if all the names appear as authors to find out.

 The Summary can be a little exhausting to read as a list. Perhaps a summary per area instead of a listing of the principles?

 In Section 2.1 White papers, what does “open context” mean? I tried looking this up in the provided citation and it still remains unclear to me.

 In Section 2 Workshop inputs, it might give a clearer picture instead of giving a paragraph to each author to instead give a paragraph to each theme they touch on. Giving space to talk to about each theme may tease apart where they agree and disagree in terms of emphasis on different aspects of openness. Thus, perhaps Section 2 could be able to provide a synthesis of all views for better comparison.

 In Section 2.2. Leader survey, why are points 4 and 5 not presented in a visual way like the other questions? A histogram or other visual aid would work well here to show support for the various items.

 In Section 4.5 Preservation and Reproducibility, I think there could be a lot more emphasis on what reproducibility means or at least more of an emphasis on how hard it is to pin down in a more general sense for all of science. Some citations here might help to get the point across that reproducibility is an umbrella term for many related concepts for different disciplines and doesn't only mean being able to rerun the same code. For example, you start the section with “Reproducibility depends on transparently documenting and sharing all data products, protocols, and computational algorithms (with source code) used in the research.” but of course that’s not the whole story. A scientific experiment can be fully documented and transparent and yet not reproducible (given the specific meaning of the word, as it can vary — see the citations I added for details on what I mean if need be).

 I cannot agree more with this sentence: “These arguments could be more successful than negative ones around lack of reproducibility (the “crisis narrative”).“ Transcending the crisis-style rhetoric seems like a sensible move.

 Another useful part is where you stress inclusivity and diversity. Why make things open if we’re just sharing them with the same old networks of privileged people?

 To conclude, I enjoyed reading this and I hope my feedback is useful.

 ***

 I have included some potential useful references.

I have read this submission. I believe that I have an appropriate level of expertise to confirm that it is of an acceptable scientific standard.

## References

[1] GuestO, Rougier: CDS NEWSLETTER - The Newsletter of the Technical Committee on Cognitive and Developmental Systems.2016;4-4 Reference source

[2] Olivia Guest: Varieties of Reproducibility in Empirical and Computational Domains. *[accessed 14th Jan 2019]*. Reference source

[3] CooperRGuestO: Implementations are not specifications: Specification, replication and experimentation in computational cognitive modeling. *Cognitive Systems Research*.2014;27: 10.1016/j.cogsys.2013.05.001 42-49 10.1016/j.cogsys.2013.05.001

# Peer review: Referee response for version 1

**DOI:** 10.5256/f1000research.19054.r41794

**Published:** 2018-12-14

**Authors:** Lucy Montgomery

**Affiliations:** 1 Centre for Culture and Technology (CCAT), Curtin University, Bentley, WA, Australia

This paper does what it says on the tin: it is a report on the perspectives and ideas generated and captured at an invitation-only workshop. As such, it presents the discussions and themes explored within the workshop, and points toward individual white papers published elsewhere. The use of references is appropriate to the form of the report. The inclusion of a survey element, which makes the perspectives of university leaders visible within the narrative, is helpful, and adds depth to the discussion.

 I would have found the document more comfortable to read if it has acknowledged the North American and Anglophone perspectives that it captures; and the limitations that this may imply. 

 Nonetheless, the report captures the energy, and the passion of the workshop participants; and points the way to important work that still needs to be done to ensure that the open knowledge potential of universities is realised.

 While I do not agree with every point made within the document, as an opinion article and a record of a series of discussions, I don't believe that it is necessary for me to do so. By making this document public via an open platform the authors are allowing for annotation and the continuation of conversations and collaborative knowledge building. I look forward to annotating the document itself and watching as differences in perspective continue to be negotiated by both the authors and a wider community as conversations about open affordances and the future of universities unfold.

I have read this submission. I believe that I have an appropriate level of expertise to confirm that it is of an acceptable scientific standard.
